# Interleukin-6 Could Be a Potential Prognostic Factor in Ambulatory Elderly Patients with Stable Heart Failure: Results from a Pilot Study

**DOI:** 10.3390/jcm10030504

**Published:** 2021-02-01

**Authors:** Marina Povar-Echeverría, Pablo Esteban Auquilla-Clavijo, Emmanuel Andrès, Francisco Javier Martin-Sánchez, María Victoria Laguna-Calle, Alberto Elpidio Calvo-Elías, Noel Lorenzo-Villalba, Manuel Méndez-Bailón

**Affiliations:** 1 Servicio de Medicina Interna, Hospital Universitario Miguel Servet, 50009 Zaragoza, Spain; marinapovar89@hotmail.com; 2 Servicio de Cardiología, Hospital Royo Villanova, 50015 Zaragoza, Spain; pabloauq50@gmail.com; 3 Service de Médecine Interne, Diabète et Maladies Métaboliques, Hôpitaux Universitaires de Strasbourg, 67000 Strasbourg, France; emmanuel.andres@chru-strasbourg.fr; 4 Servicio de Medicina Interna, Facultad de Medicina, Instituto de Investigación Sanitaria del Hospital San Carlos, Hospital Clínico San Carlos, Universidad Complutense de Madrid, 28040 Madrid, Spain; fjms@hotmail.com (F.J.M.-S.); victorialaguna69@gmail.com (M.V.L.-C.); ae.calcoelias@gmail.com (A.E.C.-E.); manuelmenba@hotmail.com (M.M.-B.)

**Keywords:** heart failure, biomarkers, inflammations, interleukin-6

## Abstract

Introduction: Inflammation is a fundamental phenomenon in heart failure, but the prognostic or therapeutic role of markers such as interleukin-6 (IL-6) has not yet been clarified. The objective of this study is to describe the clinical profile of patients with elevated IL-6 and determine if they have worse clinical outcomes. Methods: A retrospective c.ohort observational study including 78 patients with heart failure followed up at the Heart Failure Outpatient Clinic of the Internal Medicine Department. IL-6 was determined in all patients, who were then assigned into two groups according to IL-6 level (normal or high). Clinical and prognostic data were collected to determine the differences in both groups. Results: The average age was 79 years, 60% female. A total of 53.8% of the patients had elevated IL-6 (group 2). Patients with elevated IL-6 presented more frequently with anemia mellitus (64.3% vs. 41.7%; *p* = 0.046), atrial fibrillation (83.3% vs. 61.9% *p* = 0.036), dyslipidemia (76.2% vs. 58.2%; *p* = 0.03), higher creatinine levels (1.35 mg/dL vs. 1.08 mg/dL; *p* = 0.024), lower glomerular filtration rate (43.6 mL/min/m^2^ vs. 59.9 mL/min/m^2^; *p* = 0.007), and anemia 25% vs. 52.4% *p* = 0.014. The factors independently associated with the increase in IL-6 were anemia 3.513 (1.163–10.607) and renal failure 0.963 (0.936–0.991), *p* < 0.05. Mortality was higher in the group with elevated IL-6 levels (16% vs. 2%; *p* = 0.044) with a log-rank *p* = 0.027 in the Kaplan–Meier curve. Conclusion: Patients with heart failure and elevated IL-6 most often have atrial fibrillation, diabetes mellitus, dyslipidemia, anemia, and renal failure. In addition, mortality was higher and a tendency of higher hospital admission was observed in stable HF patients with elevated IL-6.

## 1. Introduction

Inflammation has been implicated in the pathogenesis of heart failure (HF). In response to metabolic stress, hemodynamic overload, and neurohormonal hyperactivation in the endothelium and myocardial cells, proinflammatory cytokines such as tumor necrosis factor-α (TNFα), and interleukin (IL) 1 and 6 are released. Initially, these markers favor the adaptation of the cardiovascular system to stress, but later in the course of the disease, they produce deleterious effects through endothelial and cardiac dysfunction leading to myocardial fibrosis [1]. Previous studies have shown an increase in inflammatory markers such as C-reactive protein (CRP), tumor necrosis factor (TNFα), and interleukin (IL) 6 in patients with HF [2].

IL-6 expression is mostly modulated by the nuclear factor kappa B (NF-KB). NF-KB proteins are maintained in the cytoplasm by their binding with inhibitory proteins (IKBs). Cytokines, infections, and toxins can induce the phosphorylation, ubiquitinization, and subsequent degradation of the IKB protein by the proteasome. This allows NF-KB to translocate to the nucleus and bind cognate DNA-binding sites to regulate the transcription of a large number of genes, including inflammatory cytokines [3].

However, the potential utility of inflammatory markers in the diagnosis, treatment, and prognosis of this syndrome has not yet been clarified. The need to clarify the potential utility of these markers in the diagnosis, risk stratification, or even therapeutic targets in heart failure has been investigated in current clinical trials. In this respect, the studies ATTACH [4] and RENEWAL [5] targeting TNFα did not have relevant clinical results in HF [6]. In contrast, the CANTOS study [7] recently demonstrated that canakinumab, a monoclonal antibody that binds and blocks interleukin IL-1, reduces the risk of major adverse cardiovascular events (MACE) without affecting lipid levels in patients with a history of acute myocardial infarction with elevated CRP. The cardiovascular benefits increased as CRP was reduced. A secondary analysis of the CANTOS study [8] found that IL-6, a pro-inflammatory cytokine normally stimulated by IL-1β, could play a fundamental role in both global and cardiovascular prognosis.

These results have renewed the interest in the potential utility of IL-6 in HF as a therapeutic target. Some studies indicate that other inflammatory markers such as copeptin and mid regional pro-adrenomedullin (MRproADM) could be elevated in patients with early readmission after an episode of acute heart failure [9]. The BIOSTAT-CHF registry that included patients with HF showed that 56% of cases had elevated IL-6 and related it to age, preserved ejection fraction, higher N-terminal prohormone of brain natriuretic peptide (NT-proBNP) concentration, lower hemoglobin concentration, and iron deficiency. In addition, the increase in IL-6 was related to an increase in mortality and readmission [6].

IL-6 levels have been reported as predictors of mortality in acute HF and acute coronary syndromes as well as severe chronic HF in some studies [10,11], but data on ambulatory elderly patients with heart failure are scarce in the current literature.

The objective of this study was to assess the potential prognostic value of IL-6 in ambulatory elderly patients with stable HF, as well as to describe the clinical profile of patients with elevated IL-6 levels.

## 2. Methods

We conducted a retrospective observational cohort study including patients diagnosed with heart failure and a follow-up in the outpatient clinic of heart failure in the Internal Medicine department of our tertiary care university hospital between 30 January 2014 and 30 April 2018. The diagnosis of heart failure was made according to the criteria of the American Heart Association guidelines 2019 [12].

The data were collected at the time of the medical visit: age and sex, blood pressure, heart rate, smoking, lifestyle, comorbidities, baseline status (Pfeiffer test, nutritional assessment, and frailty), etiology of HF, laboratory and echocardiographic data (left ventricular ejection fraction was considered as reduced if <50% or preserved ≥50% according to the American Heart Association guidelines 2019), as well as pharmacological treatment.

For the present study, IL-6 was determined once in all patients during the first medical visit. Patients with a diagnosis or suspicion of neoplasia, rheumatological disease, sepsis, pneumonia or any disease that could interfere with the IL-6 value were excluded. IL-6 was measured by electrochemiluminescence immunoassay (ECLIA) using the sandwich technique and was considered high when the value was greater than 7 pg/mL, which is the reference cut-off point of the laboratory of our center and is consistent with other studies [6]. The sample was divided right after into two groups based on the cut-off point of IL-6 (group 1: IL-6 ≤ 7 pg/mL; group 2: IL-6 > 7 pg/mL).

The follow-up was carried out by consulting the clinical history of the center, outside medical records, and administrative databases.

The main outcome variables were all-cause mortality of the health system. The latter included: hospital admission for HF, visit to the emergency department for HF, unscheduled attention in day-hospital care for heart failure decompensation, and mortality from any cause from the date of the determination of IL-6 until the occurrence of the first event and/or end of study.

Qualitative variables were presented as frequency and percentage (%) and quantitative variables as mean and standard deviation (SD) or as median and interquartile range (IQR) after performing normality tests using the Kolmogorov–Smirnov test with the Lilliefors correction. The comparison of quantitative variables between independent groups was performed using the Student’s t and Mann–Whitney U tests. The comparison between qualitative variables was made using the Chi-square and Fisher tests.

Multivariate analysis was performed using binary logistic regression, in which variables with statistical significance (*p* < 0.1) were included in the univariate analysis. Kaplan–Meier curves were constructed for the analysis of survival and for the free time to HF admission after one year of follow-up. The comparison between the groups was made with the log-rank test. In all cases, the level of statistical significance was established for a value of *p* <0.05. Statistical analysis was performed with the Statistical Package for Social Sciences program (version 21.0, SPSS Inc. Chicago, IL, USA).

## 3. Results

A total of 78 patients were included. The mean age was 79 years (SD 6.6), and 46 (60%) were female. The most frequent type of underlying heart disease was hypertensive cardiomyopathy in 32 (41.1%), with no differences between the two groups.

The characteristics of the study population and a comparison of both groups are shown in Table 1. Patients with elevated IL-6 (Group 2) were more likely to present with diabetes mellitus (64.3% vs. 41.7%; *p* = 0.046) and hypercholesterolemia (76.2% vs. 58.2% *p* = 0.03). Group 1 patients (normal IL-6) had a higher frequency of atrial fibrillation compared to Group 2 (83.3% vs. 61.9%; *p* = 0.036). In the group with elevated IL-6, anemia was more frequent (52% vs. 25%; *p* = 0.014). Regarding the laboratory variables, patients in the group with elevated IL-6 had higher creatinine levels (1.35 mg/dL vs. 1.08 mg/ dL; *p* = 0.024), with lower glomerular filtration rates (43.6 mL/min/m^2^ vs. 59.9 mL/min/m^2^; *p* = 0.007). There was no linear correlation between NT-proBNP values and IL-6.

In the sample, 64.1% of patients presented with a preserved ventricular ejection fraction versus 35.8% with reduced ejection fraction. In the group of patients with elevated IL-6 levels, 72.1% presented with a preserved ventricular ejection fraction and 28.5% reduced ejection fraction without reaching statistical significance. Also in this group, the hospitalization rate for heart failure decompensation was higher (40% vs. 22%) without significant differences (*p* = 0.85) (Table 1). A box-plot including IL-6 levels and left ventricular ejection fraction was made (Supplementary Material).

Regarding the medical treatment received, there are no differences between the two groups except for anticoagulation, which is more frequent in group 1 (86.1% vs. 59.5%; *p* = 0.009).

The factors independently associated with the increase in IL-6 were anemia 3.513 (1.163–10.607); *p* = 0.026 and GFR CKD EPI 0.963(0.936–0.991); *p* = 0.009. (Table 2)

A multivariate analysis including age, IL-6 levels, and the left ventricular ejection fraction was conducted. The IL-6 levels were independently associated to mortality in elderly patients with chronic heart failure (Table 3).

**Figure 1:**
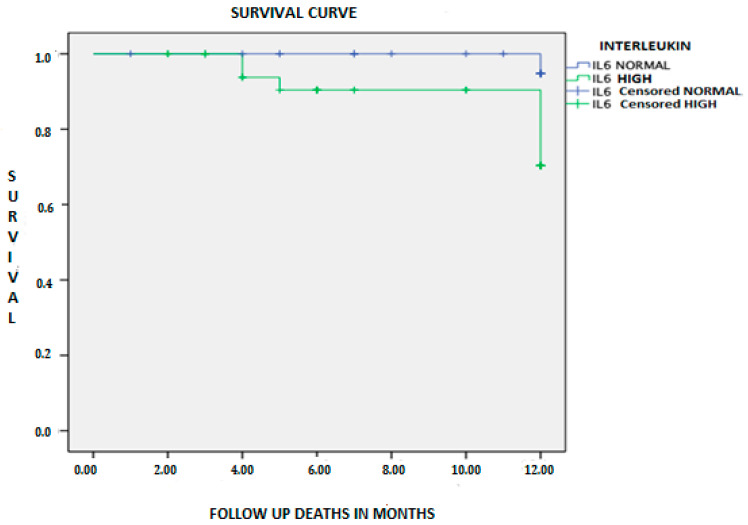
Kaplan–Meier survival curve of patients with heart failure in both groups according to interleukin-6 (IL-6) level.

The overall survival of the sample was 89.7%, with a total of 8 deaths during the follow-up period. Mortality was higher in patients with elevated IL-6 (16% vs. 2%; *p* = 0.044) (Table 1). Figure 1 shows the Kaplan–Meier survival curve for mortality during the first year, the probability of mortality being higher in the high IL-6 group (log-rank = 0.027).

**Figure 2:**
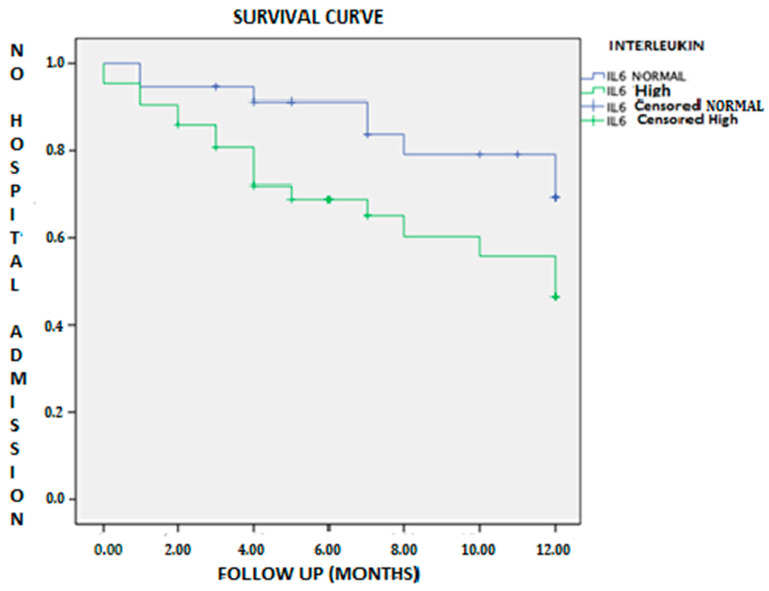
Kaplan–Meier survival curve of patients with heart failure and hospital admissions in both groups according to IL-6 level.

The probability of no hospital admission for heart failure in the first year was lower in the group with elevated IL-6 (log-rank test = 0.055); in other words, the free time of hospitalization was lower in this group (Figure 2).

The Kaplan–Meier survival curve of patients with heart failure according to IL-6 levels until the combined event (hospital admission, emergency care for HF, day hospital care for HF, and mortality) was not statistically significant (log-rank test = 0.097).

## 4. Discussion

In this retrospective study, approximately half of the patients had elevated IL-6. These patients more frequently presented with hypercholesterolemia, diabetes mellitus, and anemia, as well as worse kidney function (higher creatinine levels and lower glomerular filtration rates). Patients with elevated IL-6 had a worse clinical course with a higher risk of mortality from any cause. When multivariate analysis was performed, the variables independently associated with an increase in IL-6 were anemia and renal dysfunction.

Diabetes mellitus and hypercholesterolemia are well-established factors in the development of atherosclerosis. These conditions contribute to an inflammatory process that could explain the high levels of this marker. There are studies, such as the ADVANCE trial [13], which indicate that patients with diabetes mellitus and elevated IL-6 would have a higher risk of developing heart failure. In our study, type 2 diabetes mellitus was found in 42 patients and high IL-6 levels in 27 of them. Observational studies have shown that IL-6 is a risk factor for type 2 diabetes [14,15] but its role in beta cell survival in type 1 diabetes is small.

Anemia plays an important role in heart failure; patients with anemia have a worse prognosis and less exercise capacity [16]. It is known that IL-6 stimulates hepatocytes to produce hepcidin, which inhibits intestinal absorption and reticuloendothelial release of iron [3]. IL-6 also induces a significant increase in the expression of hepcidin mRNA, independent of IL-1 or TNF-α activity [17]. Our data are comparable to those of other series, such as the results of the BIOSTAT-CHF study [6]. In this study, patients with elevated IL-6 were more likely to present with anemia, and an elevated IL-6 was associated with increased rates of hospitalization for heart failure and mortality. Other mechanisms of anemia induced by IL-6 include rapid hemodilution, impairment of erythroid proliferation, and maturation and downregulation of the membrane-bound erythropoietin receptor [18]. Anemia has negative effects on the aerobic capacity, endurance, energetic efficiency, and work productivity.

Elevated proBNP was an independent predictor of elevated IL-6 in patients from the BIOSTAT-CHF study [6]. This could be explained as stretched cardiomyocytes and cardiac fibroblasts produce IL-6, IL-1, and TNF-𝛼. IL-6 has already been associated with diastolic dysfunction and downregulation of the expression of sarcoplasmic reticulum Ca2 + -ATPase (SERCA2) channels in cardiomyocytes and subsequently impairs the diastolic relaxation of contractile proteins [19]. Free calcium increases during systole, and it is removed from the cytosol primarily by the action of SERCA during diastolic relaxation [20]. Cardiomyocyte stiffness has also been related to IL-6 through other metabolic pathways. In the abovementioned study, mean age was 69 ± 12 years, 74% patients were male, and predominantly presented with heart failure with reduced ejection fraction in contrast to our study in which mean age was 79 years, 60% were female, and 50% of patients presented with preserved left ventricular ejection fraction. In contrast to their results, no relation was found between the NT-proBNP levels and IL-6 in our study, which could probably be due to the fact that the majority of patients presented with preserved left ventricular ejection fraction, and they tend to have lower NT-proBNP values than patients with reduced ventricular ejection fraction.

In some studies, it has been suggested that elevated IL-6 could be involved in the pathogenesis of cardiorenal syndrome (CRS) [6,21], since patients with elevated IL-6 had lower glomerular filtration figures, greater neurohormonal activation, diuretic resistance, and mortality. Although in our study we have not measured other indicators of CRS such as resistance to diuretics, we have also observed worse kidney function with higher creatinine levels and lower glomerular filtration rate in patients with elevated IL-6. According to our results, IL-6 could be an independent factor associated with mortality in elderly patients with chronic heart failure despite model adjustment by age and left ventricular ejection fraction, which are well-known factors associated with poor prognosis and mortality in this entity. There is strong evidence that IL-6 serum concentration increases with age [22,23,24,25,26,27,28,29]. In elderly individuals, increments in IL-6 levels are not explained by differential prevalence of IL-6 gene polymorphisms; however, there are available data supporting that the excessive production or reduced clearance of oxygen-free radicals may stimulate IL-6 production and play an important role [30]. Some previous studies have reported a significant increase of sIL-6r up to the seventh decade followed by a gradual decline [31]. Disturbances in the IL-6 trans-signaling have been reported with aging; these may lead to a reduction of circulating soluble glycoprotein 130, a soluble receptor that acts as an inhibitor of IL-6 function [32].

However, findings are inconsistent, so further investigations are necessary to understand the effect of age on circulating levels of sgp130 and sIL-6r. Furthermore, some studies have demonstrated an independent association between higher IL-6 and younger age in patients with heart failure [6]. In the BIOSTAT-CHF study, an independent association between higher IL-6 and younger age was reported. The changes in the body composition in elderly patients are well known, and that is also another important correlate of IL-6, especially the percentage of visceral fat. It is considered that the IL-6 produced by omental adipose tissue accounts for 10% to 35% of the body’s basal circulating IL-6 level [33]. IL-6 levels have been already described as predictors of mortality in acute HF and acute coronary syndromes as well as chronic HF in some studies. One of these studies included 102 patients with severe CHF (New York Heart Association class III to IV) admitted to the hospital [10], and the other was a prospective longitudinal study of 75 patients with the diagnosis of AHF and/or ACS [11] in contrast to our study performed in ambulatory and stable patients. Our results are in consonance with the BIOSTAT-CHT study, but in their cohort, mean age was 69 ± 12 years versus 79.2 ± 6.6 in ours.

In our study, 70% of the patients had atrial fibrillation (AF). It is known that AF is an important prognostic marker in patients with HF. There are studies that show that adequate treatment of AF improves the prognosis of HF. The CASTLE-AF study compares the standard treatment of AF with the ablation of persistent or paroxysmal AF in patients with HF and ventricular dysfunction, observing a reduction in all-cause mortality and readmissions for HF in the catheter ablation group with statistically significant differences [34].

Several studies have described the increase in CRP and IL-6 levels in patients with paroxysmal and permanent AF and suggest that the presence of inflammation promotes the development or perpetuates arrhythmia. Although the mechanism has not been fully clarified, it appears that the fibrotic changes stimulated by the inflammation cascade in the atrium could be responsible. These data are supported by the finding of inflammatory infiltrates with the presence of inflammatory cytokines (IL-1, IL-6, and TNFα), myocyte necrosis, and fibrosis in atrial biopsies of patients with AF. Furthermore, fibrotic changes in the left atrium have been shown to be an important link between atrial fibrillation and the development of heart failure [35,36,37]. There are even studies that indicate that left ventricular fibrosis quantified by T1 mapping using cardiac MRI can be a potential predictor of adverse events in patients with HF and coexisting AF [38].

Despite all the abovementioned elements, in our study, the group with elevated IL-6 had a lower proportion of AF. However, the study follow-up period was short and assessing new onset AF was not one of the objectives of this work. A long-term follow-up study would be of help to assess what proportion of patients with IC and elevated IL-6 develops AF.

Our study has certain limitations. First, it is a retrospective design, which implies a possible bias in obtaining information. As it is a study carried out in a single center, the characteristics of the patients could limit the generalizability of the results; however, the sample represents the phenotype of patients with heart failure follow-up in the internal medicine departments in Spain. This is a pilot study with a limited sample size, which may have limited the power of the study. Left ventricular ejection fraction was considered reduced if <50% or preserved ≥50% and not as HFrEF, HFmrEF, and HFpEF, which could be interesting to analyze in further studies. A large cohort study will be needed to confirm our results. We would like to highlight the lack of data about stable elderly patients with heart failure in the current medical literature.

We consider our study of particular interest given the role that IL-6 plays in the pathogenesis of HF, as well as its potential usefulness as a marker that identifies patients at risk of mortality. Patients with elevated interleukin-6 seem to have a special phenotype with a greater association with cardiorenal syndrome and anemia, both important comorbid conditions associated with heart failure.

## 5. Conclusions

IL-6 was elevated in a subgroup of patients with heart failure, dyslipidemia, atrial fibrillation diabetes mellitus, anemia, and chronic renal failure as already described. In this respect, anemia, chronic renal failure, and atrial fibrillation were the conditions independently associated with elevated IL-6 levels. Therefore, in our study, mortality was higher, and we observed a tendency of higher hospitalization in stable HF patients with elevated IL-6.

## Figures and Tables

**Table 1:** Characteristics of the study population and comparison between both groups.

	Total (N = 78)	IL-6 Normal (N = 36)	IL-6 High (N = 42)	*p*
Baseline characteristics				
Age in years—median (SD)	79.2 ± 6.6	78.5 ± 5.3	79.8 ± 7.5	0.374 ^a^
Sex female—*n* (%)	46 (59)	21 (58.3)	25 (59.5)	0.915 ^b^
Smoking	4 (5.1)	2 (5.6)	2 (4.8)	0.874 ^b^
Sedentary	48 (61.5)	21 (58.3)	27 (64.3)	0.590 ^b^
Underlying cardiomyopathy				
Hypertensive cardiomyopathy	32 (41.1)	17 (47.2)	15 (35.7)	0.303 ^b^
Ischemic cardiomyopathy	17 (21.8)	5 (13.9)	12(28.6)	0.117 ^b^
Valvulopathy	20 (25.6)	10 (27.8)	10 (23.8)	0.689 ^b^
Nonischemic dilated cardiomyopathy	6 (7.7)	3 (8.3)	3 (7.1)	0.844 ^b^
Cor pulmonale	3 (3.8)	1 (2.8)	2 (4,8)	0.65 ^b^
Comorbidities				
Arterial hypertension	78(100)	36 (46)	42 (54)	0.521 ^b^
Diabetes mellitus	42 (53.8)	15 (41.7)	27 (64.3)	**0.046** ^b^
Hypercholesterolemia	51 (65.4)	19 (52.8)	32 (76.2)	**0.030** ^b^
Metabolic syndrome	24 (30.8)	8 (22.2)	16 (38.1)	0.130 ^b^
Atrial fibrillation	56 (71.8)	30 (83.3)	26 (61.9)	**0.036** ^b^
Chronic renal failure	31 (39.7)	15 (41.7)	16 (38.1)	0.748 ^b^
Anemia	31 (39.7)	9 (25)	22 (52.4)	**0.014** ^b^
Obesity (BMI >30)	17 (21.8)	9 (25)	8 (19)	0.526 ^b^
Functional evaluation				
Pfeiffer Test				
0-2 mistakes	73 (93.6)	33 (91.7)	40 (95.2)	0.465 ^b^
>2 mistakes	5 (6.4)	3 (8.3)	2 (4.8)	
Nutritional evaluation (MNA Test)				
Normal (≥24 points)	52 (66.7)	25 (75.8)	27 (67.5)	0.438 ^b^
At risk of malnutrition or established malnutrition (<24 points)	21 (26.9)	8 (24.2)	13 (32.5)	
Frailty (Barber >1)	71 (91)	32 (88.9)	39 (92.9)	0.541 ^b^
Echocardiographic parameters				
Left ventricular hypertrophy	47 (60.3)	21 (58.3)	26 (61.9)	0.648 ^b^
Preserved LVEF (≥50%)	50 (64.1)	20 (55.6)	30 (71.4)	0.145^b^
Reduced LVEF (<50%)	28(35.8)	16 (44.4)	12 (28.6)	0.163 ^d^
Vital parameters				
SBP (mmHg)	136 ± 21	136 ± 19	135 ± 23	0.230
DBP (mmHg)	68 ± 10	70 ± 9	66 ± 10	0.697
Heart rate (beats/min)	78 ± 16	77 ± 17	78 ± 16	0.582
Blood results				
Hemoglobin (g/dl)	12.7 ± 1.9	13.2 ± 2.3	12.3 ±2.1	**<0.001**
Ferritin (ng/mL)	175 ± 289	147 ± 223	194 ± 322	0.108 ^c^
Creatinine (mg/dl)	1.28 ± 0.65	1.08 ± 0.57	1.35 ± 0.69	**0.024**
CKD-EPI glomerular filtration rate (mL/min/m^2)^	46 ± 26	59.95 ± 35	43.6 ± 19	**0.007**
Microalbuminuria (mg/L)	16.9 ± 60.7	15.9 ± 25.8	17.6 ± 99	0.451 ^c^
HbA1c (%)	6 ± 1	6 ± 1	6.3 ± 1	0.108 ^c^
NT-ProBNP (pg/mL)	1613 ± 2297	1244 ± 2780	1990 ± 4019	0.061 ^c^
Usual treatment				
Beta Blockers	52 (66.7)	21 (58.3)	31 (73.8)	0.148 ^b^
Furosemide	65 (83.3)	31 (86.1)	34 (81)	0.542 ^b^
Chlorthalidone	6 (7.7)	5 (13.9)	1 (2.4)	0.057 ^b^
Spironolactone/Eplerenone	33 (42.3)	13 (36.1)	20 (47.6)	0.305 ^b^
ACE inhibitors/Angiotensin II Antagonist	23 (29.5)	13 (36.1)	10 (23.8)	0.235 ^b^
Statins	52 (66.7)	23 (63.9)	29 (69)	0.63 ^b^
Anticoagulation	56 (71.8)	31 (86.1)	25 (59.5)	**0.009** ^b^
Follow-up				
Event (combined)	40 (51.3)	15 (41.7)	25 (59.5)	0.116 ^b^
HF hospitalization	25 (32.1)	8 (22.2)	17 (40.5)	0.069^d^
Visit to ED for HF	13 (16.7)	4 (11.1)	9 (21.4)	0.223 ^b^
Visit to Day Care Hospital for HF	11 (14.1)	6 (16.7)	5 (11.9)	0.547 ^b^
Mortality from any cause	8 (10.3)	1 (2.8)	7 (16.7)	**0.044** ^b^

BMI: body mass index; MNA: Mini nutritional assessment; LVEF: left ventricular ejection fraction; SBP: Systolic blood pressure; DBP: diastolic blood pressure; HbA1c: Glycosylated hemoglobin; NT-ProBNP: N-terminal prohormone of brain natriuretic peptide; ACE inhibitors: Angiotensin-converting enzyme inhibitors; HF: heart failure; ED: Emergency department. Data presented as mean and standard deviation (SD), median and interquartile range (QR), or number (percentage). Median and standard deviation (SD): age, SBP, DBP; median and interquartile range (QR): Ferritin, Microalbuminuria, HbA1c, ProBNP. Student’s T test was used to compare quantitative variables between independent groups (parametric variables tended to normality). Mann–Whitney U tests were used to compare quantitative variables between independent groups (parametric variables do not tend to normality). Chi-squared and Fisher test were used to compare qualitative variables. ^a^ T Student; ^b^ Chi squared; ^c^ Mann–Whitney U test, and ^d^ Fisher test.

**Table 2:** Factors associated with increased interleukin-6 (IL-6) levels.

	Multivariable OR (95%CI)	*p*
Atrial fibrillation	1.240 (0.332–4.626)	0.749
Diabetes mellitus	0.41 (0.128–01.134)	0.134
Anemia	3.513 (1.163–10.607)	0.026
Hypercholesterolemia	0.565 (0.177–1.802)	0.335
GFR CKD EPI	0.963 (0.936–0.991)	0.009

Odds ratio and *p*-values are presented; the values within parentheses are 95% confidence intervals and *p* value < 0.05.

**Table 3:** Logistic regression analysis of factors associated to mortality.

	Multivariable	
Factors Associated with Mortality	OR (95%CI)	*p*
Age	1.131 (0.992–1.290)	0.066
Left ventricular ejection fraction	0.207 (0.018–2.393)	0.207
IL-6 levels	1.037 (1.000–1.074)	0.048

Odds ratio and *p*-values are presented; the values within parentheses are 95% confidence intervals, and *p* value < 0.05.

## Data Availability

Not applicable.

## Supplementary Materials

The following are available online at https://www.mdpi.com/2077-0383/10/3/504/s1, Figure S1: Box-plot including IL-6 levels and left ventricular ejection fraction.
