# Supplementary figures and images for: Interleukin-6 Could Be a Potential Prognostic Factor in Ambulatory Elderly Patients with Stable Heart Failure: Results from a Pilot Study
Source: J Clin Med. 2021 Feb 1;10(3):504. doi: 10.3390/jcm10030504 (PMC7867065; doi:10.3390/jcm10030504)

Supplementary material

Box-plot including IL-6 levels and left ventricular ejection fraction

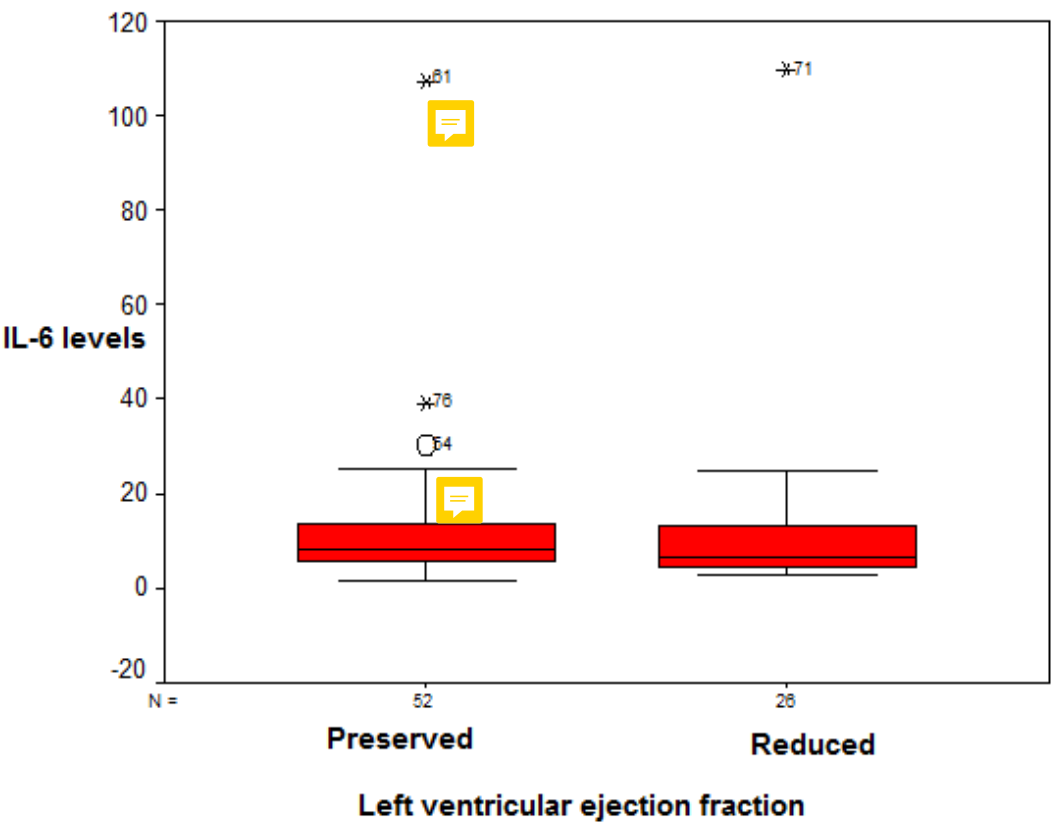

Supplement: Supplementary file 1 [file jcm-10-00504-s001.pdf]
